# Cooling Subgrade Effectiveness by L-Shaped Two-Phase Closed Thermosyphons with Different Inclination Angles and XPS Insulation Boards in Permafrost Regions

**DOI:** 10.3390/ma15238470

**Published:** 2022-11-28

**Authors:** Yalong Zhou, Xu Wang, Chunxiang Guo, Yuan Hu, Fei He, Deren Liu, Daijun Jiang

**Affiliations:** 1 School of Civil Engineering, Lanzhou Jiaotong University, Lanzhou 730070, China; 2 National and Provincial Joint Engineering Laboratory of Road & Bridge Disaster Prevention and Control, Lanzhou 730070, China; 3 Key Laboratory of Road & Bridge and Underground Engineering of Gansu Province, Lanzhou 730070, China

**Keywords:** permafrost, composite subgrade, L-shaped TPCT, XPS insulation board, coupled heat transfer model, cooling effectiveness

## Abstract

This study focused on the coupling heat transfer mechanism and the cooling efficiency of L-shaped two-phase closed thermosyphons (L-shaped TPCTs) in the wide subgrade of permafrost regions. Considering the fact that time–space dynamics change the effects of the air temperature, wind speed, and geotemperature, a coupled air temperature–L-shaped TPCT–subgrade soil heat transfer model was established using the ANSYS 15.0 software platform, and the rationality of the model was verified through measured data. The heat-transfer characteristics of the L-shaped TPCTs and the long-term thermal stability of the subgrade were studied under different inclination angles of the evaporator (α = 15°, 30°, 50°, 70°, and 90°). Then, the cooling effectiveness of a composite subgrade with TPCTs and an XPS insulation board was numerically calculated. The results show that the heat flux of the L-shaped TPCT was the greatest when α = 50°, and the heat flux reached the maximum value of 165.7 W·m^−2^ in January. The L-shaped TPCT had a relatively good cooling effect on the subgrade as a whole when α = 50° and 70°, but the thawing depth at the center of the subgrade with L-shaped TPCTs reached 9.0 m below the ground surface in the 30th year. The composite subgrade with L-shaped TPCTs/vertical TPCT/XPS insulation board is an effective method to protect the permafrost foundation and improve the long-term thermal stability of the wide subgrade. The maximum heat flux of evaporation section of the L-shaped TPCT is 18.8% higher than that of the vertical TPCT during the working period of the TPCTs of the composite subgrade.

## 1. Introduction

The Qinghai–Tibet Plateau is characterized by a low latitude, high altitude, and strong solar radiation; therefore, the proposed Qinghai–Tibet expressway crosses extensive permafrost regions [1,2,3]. The wide subgrade of the expressway has a large heat storage capacity. After its construction, the strong heat absorption of black asphalt pavement will lead to a “heat gathering effect” in the center of the subgrade, which will cause the permafrost below the subgrade to melt rapidly [3,4,5]. Therefore, under the influence of engineering activities and global warming, a technique for ground heat control to meet the demands of the long-term thermal stability of the subgrade is an important issue in the construction of expressways in permafrost regions.

The TPCT is a wickless heat transfer device with a highly efficient energy-transfer capacity, and it requires no external power supply. The XPS insulation board has low thermal conductivity, high compression resistance, and high aging resistance. The TPCT and XPS insulation board have been widely used in permafrost regions to ensure thermal stability, such as the Qinghai–Tibet railway, Qinghai–Tibet highway, Qinghai–Tibet power transmission line, and China–Russia oil pipeline [6,7,8,9,10,11,12]. TPCTs are usually inserted vertically into a subgrade at the shoulders or toes along the subgrade direction in permafrost regions. The soil near the shoulder has a good cooling effect under this measure, but this cooling effect on the soil at the center of the subgrade is small [13,14]. In recent years, to expand the cooling range of the wide subgrade, effectively eliminate the “heat gathering effect” of the road substrate, and ensure the uniform and symmetrical distribution of the temperature field of the wide expressway subgrade in the permafrost regions, researchers have designed inclined or curved gravity TPCTs to expand the cooling range of the foundation [15,16,17,18,19].

The research results of Mozumder et al. [20], Sarmasti et al. [21], and Cao et al. [22] have shown that the flow state of the working medium inside the TPCT is extremely complex, and the heat transfer performance of the TPCT is affected by many factors, such as the geometric shape (diameter, length, and shape), working fluid, filling ratio, wall material, radiator surface area, and inclination angle. Zhang et al. [23] found that the total thermal resistance of the inclined TPCT is at a minimum when the inclination angle is 20° and a maximum when the inclination angle is 80° through indoor tests; Yang et al. [24] obtained that, when the inclination angle of the TPCT of the Qinghai–Tibet railway is 25°–30°, the lifting effect of the permafrost table at the center of subgrade, shoulder, and slope toe is the best. Pei et al. [25] studied the cooling effect of L-shaped TPCTs on the subgrade when the evaporator section is 45°. However, the cooling efficiency of the L-shaped TPCT in the permafrost subgrade is related not only to the design parameters of the TPCT and the inclination angles of the evaporator section, but also to the actual subgrade geometry and frozen soil environment. Therefore, it is necessary to study the coupling heat-transfer characteristics of L-shaped TPCTs in a wide subgrade under different inclination angles and its cooling effectiveness on subgrade soil, along with its influence on the long-term thermal stability of the wide subgrade with L-shaped TPCTs and the XPS insulation board.

From this perspective, this paper considers the time–space dynamic change effects of the air temperature, wind speed, and the geotemperature, establishing a coupled air temperature–L-shaped TPCT–subgrade soil heat transfer model. The different inclinations (*α* = 15°, 30°, 50°, 70°, and 90°) of the evaporator section of L-shaped TPCTs and the geotemperature distribution of the subgrade are calculated and analyzed. The long-term thermal stability of the wide subgrade with the L-shaped TPCTs/vertical TPCT/XPS insulation board system in permafrost regions was evaluated.

## 2. Heat Transfer Model of the Subgrade with L-Shaped TPCTs

### 2.1. Heat Conduction Equation of Soil

Considering the heat conduction of the soil skeleton and water, along with the frozen soil phase change, the differential equation of heat conduction in the soil is as follows [26,27]:

(1) $$\rho C\frac{\partial T}{\partial t}=\frac{\partial }{\partial x}(\lambda \frac{\partial T}{\partial x})+\frac{\partial }{\partial y}(\lambda \frac{\partial T}{\partial y})+\frac{\partial }{\partial z}(\lambda \frac{\partial T}{\partial z}),$$

where *T* is the soil temperature (°C), *ρ* is the soil density (kg·m^−3^), *λ* is the thermal conductivity of soil (W·m^−1^·°C^−1^), *C* is the specific heat capacity of the soil (J·kg^−1^·°C^−1^), and *t* is the time (s).

The sensible heat capacity method is *used* to simulate the frozen soil phase change. Assuming that the soil phase-change temperature range is (*T*_m_ ± Δ*T*), the simplified equivalent thermal conductivity and specific heat capacity can be expressed as

(2) $$\lambda_{e}=\left\{\begin{array}{l} \lambda_{f}\\ \lambda_{f}+\frac{\lambda_{u}-\lambda_{f}}{2\Delta T}[T-(T_{m}-\Delta T)]\\ \lambda_{u} \end{array}\right.\begin{array}{l} T<T_{m}-\Delta T\\ T_{m}-\Delta T\leq T\leq T_{m}+\Delta T\\ T\geq T_{m}+\Delta T \end{array},$$

(3) $$C_{e}=\left\{\begin{array}{l} C_{f}\\ \frac{L_{s}}{2\Delta T}+\frac{C_{f}+C_{u}}{2}\\ C_{u} \end{array}\right.\begin{array}{l} T<T_{m}-\Delta T\\ T_{m}-\Delta T\leq T\leq T_{m}+\Delta T\\ T\geq T_{m}+\Delta T \end{array},$$

where the subscripts f and u represent the frozen and unfrozen state of soil, respectively. *C*_e_ and *λ*_e_ are the equivalent thermal conductivity and specific heat capacity of soil, respectively. *L*_s_ is the latent heat of soil phase change.

### 2.2. Coupled Air Temperature—L-Shaped TPCT—Subgrade Soil Heat Transfer Model

**Figure 1:**
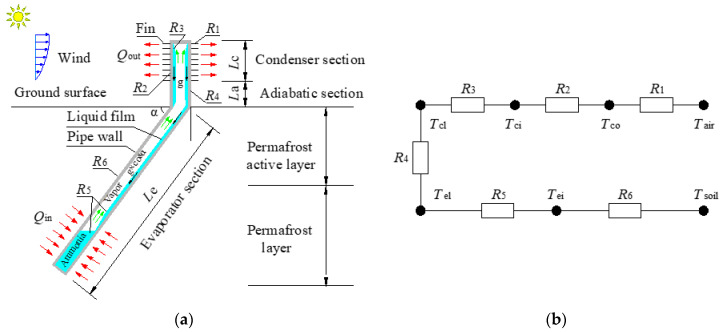
(**a**) Working mechanism diagram and (**b**) thermal resistance network of an L-shaped TPCT.

Figure 1 shows the working mechanism diagram and thermal resistance network of an L-shaped TPCT. Ammonia is used as the working medium for the TPCT, and the starting temperature difference is 0.5 °C. The length of the condensation section (*L*_c_) is 1.20 m, the length of the adiabatic section (*L*_a_) is 0.80 m, and the length of the evaporator section (*L*_e_) is 8.48 m. The inner diameter (*d*_i_) and outer diameter (*d*_o_) of the pipe are 0.057 m and 0.064 m, respectively. The fin height (*b*_n_), the fin space (*s*_n_), and the fin thickness (*δ*) are 0.025 m, 0.01 m, and 0.0015 m, respectively. The thermal conductivity coefficient of the pipe (λ) is 48 W·m^−1^·°C^−1^.

The equivalent thermal resistance of an L-shaped TPCT can be calculated using Equations (4)–(9) [25,28,29]. Table 1 shows the physical parameters of air [25], and Table 2 shows the physical parameters of the working medium of the TPCT [28,29]. *n* is the number of fins, and *r*_1_ and *r*_2_ are the outer diameters of the smooth pipe and fins, respectively. *A*_c1_ is the superficial area of the condenser section without fins, *A*_c2_ is the superficial area of the condenser section with fins, and *A*_c3_ is the total superficial area of the smooth condenser section. *d*_co_ and *d*_ci_ are the outer diameter and inner diameter of the condenser section, whereas *d*_eo_ and *d*_ei_ are the outer diameter and inner diameter of the evaporator section. *V* is the wind speed. *η* is the heat transfer efficiency of the fins. *Re*_a_ and *Pr*_a_ are the Reynolds number and Prandtl number of the flow through the outside surface of the condenser, respectively. λ_a_, *ρ*_a_, *c*_a_, and *v* are the thermal conductivity, density, specific heat at a constant pressure, and dynamic viscosity of air, respectively. *ρ*_l_, λ_l_, *c*_pl_, *L*_r_, *μ*, and *ρ*_va_ are the density, thermal conductivity, volumetric heat capacity, latent heat, dynamic viscosity, and density of the working medium, respectively. *p*_sat_ and *p*_a_ are the saturated and standard atmospheric pressure, respectively. *g* is the acceleration due to gravity. *α* is the angle of inclination between the evaporator section and the horizontal direction.

The equivalent thermal resistance of an L-shaped TPCT can be calculated as follows:

(1) Condenser section

*R*_1_ is the thermal resistance between the air and the outer wall of the condenser section [25,28]:

(4) $$R_{1}=\frac{1}{A_{c3}h_{co}},$$

where $A_{c1}=\pi d_{co}(L_{c}-n\delta )$, $A_{c2}=\pi [2n(r_{2}^{2}-r_{1}^{2})+2nr_{2}\delta ]$, $A_{c3}=\pi d_{co}L_{c}$, $h_{co}=h_{a}\frac{A_{c1}+\eta A_{c2}}{A_{c3}}$, and $h_{a}=0.1378\frac{\lambda_{a}}{d_{\mathrm{co}}}{Re}_{a}^{0.718}Pr_{a}^{\frac{1}{3}}{\left(\frac{s_{n}}{b_{n}}\right)}^{0.296}$. 

*R*_2_ is the thermal resistance for the tube wall of the condenser section [25,28]:

(5) $$R_{2}=\frac{1}{2\pi \lambda L_{c}}\ln(d_{co}/d_{ci}).$$

*R*_3_ is the thermal resistance for the liquid film formed on the inner condenser section [25,28]:

(6) $$R_{3}=\frac{1}{A_{ci}h_{ci}},$$

where $A_{\mathrm{ci}}=\pi d_{\mathrm{ci}}L_{c}$, and $h_{ci}=0.925{\left(\frac{\lambda_{l}^{3}\rho_{l}^{2}gL_{r}}{\mu q_{c}L_{c}}\right)}^{1/3}$.

(2) Adiabatic section

*R*_4_ is the thermal resistance of the adiabatic section. When the TPCT is working, *R*_4_ = 0. When the TPCT stops working, *R*_4_ = +∞.

(3) Evaporator section

*R*_5_ is the thermal resistance for the liquid film and liquid pool in the evaporator [25,28]:

(7) $$R_{5}=\frac{1}{A_{ei}h_{e}},$$

where $A_{ei}=\pi d_{\mathrm{ei}}L_{e}$, and $h_{e}=0.32\left(\frac{\rho_{l}^{0.65}\lambda_{l}^{0.3}c_{pl}^{0.7}g^{0.2}q_{e}^{0.4}}{\rho_{va}^{0.25}L_{r}^{0.4}\mu^{0.1}}\right)\cdot {\left(\frac{p_{sat}}{p_{a}}\right)}^{0.3}$.

*R*_6_ is the thermal resistance for the tube wall in the evaporator [25,28]:

(8) $$R_{6}=\frac{1}{2\pi \lambda L_{e}}\ln(d_{\mathrm{eo}}/d_{ei}).$$

Combined with the above formulas, the total effective thermal resistance can be obtained using the following formula [25,28]:

(9) $$\sum R_{i}=R_{1}+R_{2}+R_{3}+R_{4}+R_{5}+R_{6}.$$

**Figure 2:**
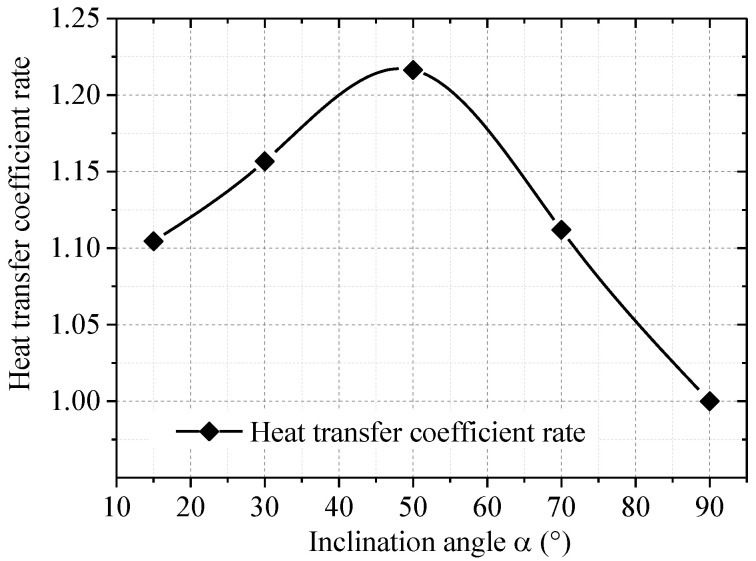
Heat transfer coefficient rate at different inclination angles of the evaporator.

The heat transfer efficiency of a TPCT is known to be closely related to its inclination angle; the condensation section of an L-shaped TPCT is vertical, and its evaporator section is inclined. Considering the tilt effect of the evaporator section, we calculated the equivalent heat transfer coefficient rate of the evaporator at different inclination angles on the basis of a prior research experiment [30,31], and the results are presented in Figure 2. This “equivalent heat transfer coefficient rate” is utilized for calculating the heat transfer coefficient of the L-shaped TPCT at different inclination angles.

Consequently, when the temperature difference between the evaporator and condenser section is greater than or equal to 0.5 °C, the total heat flow *Q* of an L-shaped TPCT is as follows [31,32]:

(10) $$Q=\frac{T_{a}-T_{co}}{R_{1}}=\frac{T_{\mathrm{co}}-T_{ci}}{R_{2}}=\frac{T_{\mathrm{ci}}-T_{cl}}{R_{3}}=\frac{T_{\mathrm{cl}}-T_{el}}{R_{4}}=\frac{T_{\mathrm{el}}-T_{ei}}{R_{5}}=\frac{T_{\mathrm{ei}}-T_{s}}{R_{6}}=\frac{T_{a}-T_{s}}{\sum R_{i}},$$

where *T*_s_ is the soil temperature around the evaporator section, and *T*_a_ is the atmospheric temperature.

According to the heat balance theory, the heat absorbed by the evaporator section of the TPCT is equal to the heat lost by the condenser section when the heat loss is ignored. Therefore, the coupled air temperature–L-shaped TPCT–soil heat transfer model can be formalized as follows [31,32]:

(11) $$\frac{T_{s}-T_{a}}{\pi d_{o}l_{e}\sum R_{i}}=\lambda_{s}\frac{\partial T}{\partial n},$$

where *λ*_s_ is the thermal conductivity of soil.

## 3. Finite Element Calculation Model and Verification

### 3.1. Model Establishment and Boundary Conditions

**Figure 3:**
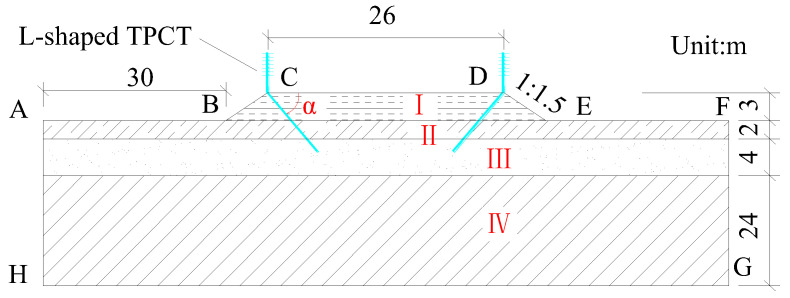
Calculation model of the subgrade with L-shaped TPCTs.

On the basis of the meteorological conditions and engineering geological conditions in the permafrost regions of the Qinghai–Tibet Plateau, and referring to the relevant specifications for the design of high-grade highways in China [33], a finite element calculation model of subgrades for the wide high-grade highway with L-shaped TPCTs in permafrost regions was established using the ANSYS finite element software, as shown in Figure 3. In the model, part I is subgrade fill, part II is gravelly sand, part III is the clayey loam layer, and part IV is strongly weathered mudstone. The thermophysical parameters of different soils are shown in Table 3 [25].

According to the observation data for the Qinghai–Tibet Plateau over many years, and considering the effects of global warming, the annual average air temperature will increase by about 2.6 °C in the coming 50 years. The temperature boundary conditions are simplified according to the boundary layer theory, and the simplified expressions of the temperature variations are given in Equation (6) and Table 4.

(12) $$T_{a}=T_{0}+A\sin(\frac{2\pi }{8760}t+\frac{\pi }{2}+\alpha_{0})+\frac{2.6\times t}{50\times 365\times 24},$$

where *T*_0_ is the mean annual ground temperature, *A* is the annual amplitude of temperature, *t* is the time in hours, and *α*_0_ is the phase angle, which is determined by the finishing time of the subgrade.

According to the measured wind speed in the Qinghai–Tibet Plateau, the wind speed at the height *H* above the ground can be calculated using the following formula [28]:

(13) $$V=(3.64+1.10\sin(\frac{2\pi }{8760}t+\frac{3\pi }{2}+\alpha_{0})){\left(\frac{H}{10}\right)}^{0.16},$$

where *t* is the time in hours, *H* is the height above the ground (m), and *α*_0_ is the phase angle.

The left and right boundaries (AH and FG) of the computational model are assumed to be adiabatic, and a constant heat flux of *q* = 0.03 W·m^−2^ is applied to the bottom boundary (HG) of the computational model. The heat flux of the evaporator section of the TPCT is applied to the model node in the form of linear heat flux.

In this study, we assumed that the subgrade was constructed on July 15 (α_0_ = 0). The initial temperature distributions of the natural foundation were obtained through a long-term transient solution with the boundary condition without considering global warming. Furthermore, it was found that the initial temperatures of the subgrade are dependent on the actual temperature of the natural ground surface. The APDL language in ANSYS was used to compile corresponding programs to realize the calculation of the coupled air temperature–L-shaped TPCT–subgrade soil heat transfer model.

### 3.2. Model Validation

**Figure 4:**
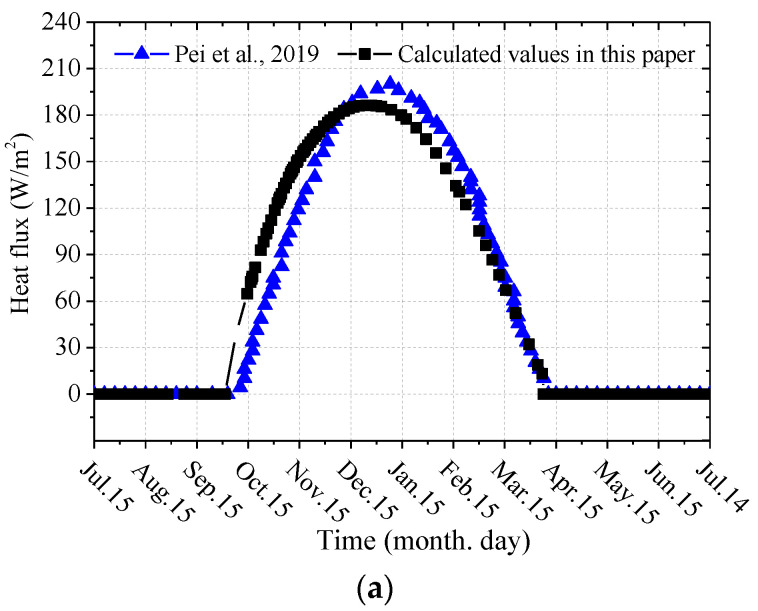

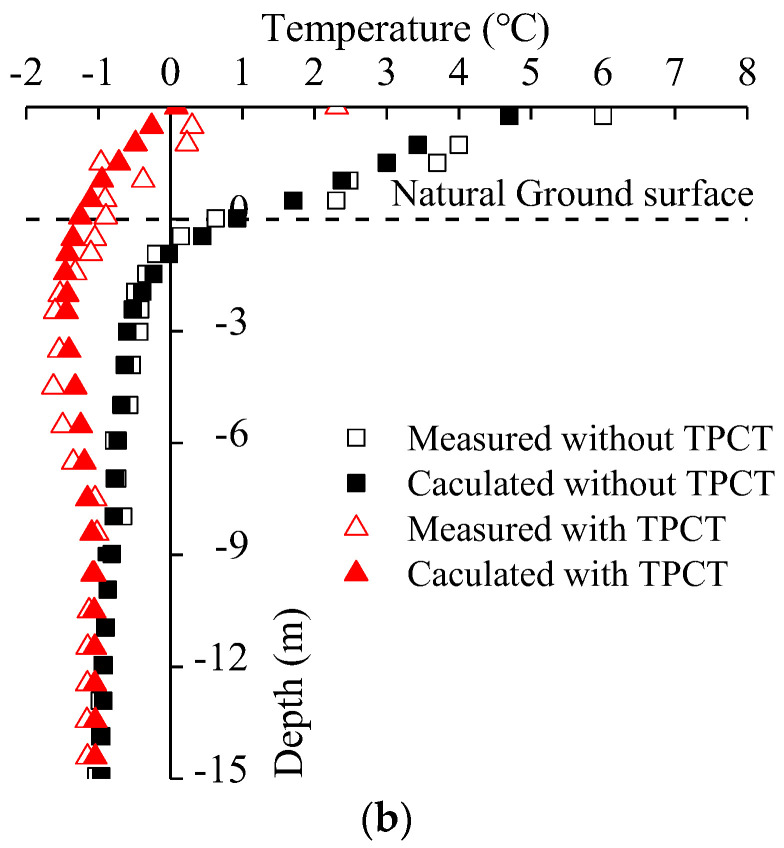
Validation of the numerical calculation results: (**a**) heat flux of the TPCT (fifth year) [25]; (**b**) geotemperatures at the of subgrade center (15 October, fifth year).

In permafrost regions, the L-shaped TPCT has not been tested in the subgrade of a wide expressway, and there is still a lack of field data to verify the established model. However, the coupled model of a wide subgrade with an L-shaped TPCT established above is universal and can simulate various working conditions through changes in the boundary conditions, geological conditions, and model size. Pei et al. [25] carried out a field test on a subgrade with an L-shaped TPCT (*α* = 45°) in Bailu River, Qinghai–Tibet Plateau of China; the geological conditions of the test site, the geometric dimensions of the subgrade, the frozen soil environment, and the L-shaped TPCT parameters were detailed in a previous study [25]. On the basis of field tests, the coupled model of the subgrade with L-shaped TPCTs was established using the ANSYS software, where the thermophysical and mechanical parameters of soil and the boundary conditions of the model were selected according to the numerical calculations found in previous literature [25]. To verify the rationality of the aforementioned models and calculation results, the calculated values of heat flux at the outer walls of the evaporator sections and the ground temperature at the centerline of the subgrade were selected and compared with the field-measured values, as shown in Figure 4.

Figure 4a shows the comparison curves between the heat flux calculated by Pei et al. [26] and the value calculated in this paper. It can be seen from the figure that the change trend of the two curves remained basically consistent. The calculation results of the working start time, the working end time, and the change in the heat flux with time during the working period of the L-shaped TPCT in this paper are in good agreement with the findings of Pei et al. [25]. Figure 4b shows the comparison curves of the measured and calculated ground temperature at the subgrade center. It can be seen from the figure that the numerical simulation value of the ground temperature along the depth of the subgrade was basically consistent with the measured value. Only the surface near the subgrade had a relatively large error, which may have been due to the influence of ambient temperature, light, and other factors. On the basis of the heat-transfer characteristics of the L-shaped TPCT and the comparison of the ground temperature of the subgrade, it can be concluded that the coupled heat transfer model can better predict the long-term thermal stability of the subgrade with L-shaped TPCTs in permafrost regions.

## 4. Influence of Evaporator Section Inclinations on the Cooling Effectiveness

The calculation models of an ordinary subgrade (no TPCT) and five wide subgrades with L-shaped TPCTs (*α* = 15°, 30°, 50°, 70°, and 90°) were established. The heat-transfer characteristics of L-shaped TPCTs with different inclinations and the long-term thermal stability were analyzed.

### 4.1. Heat-Transfer Characteristics of L-Shaped TPCTs

**Figure 5:**
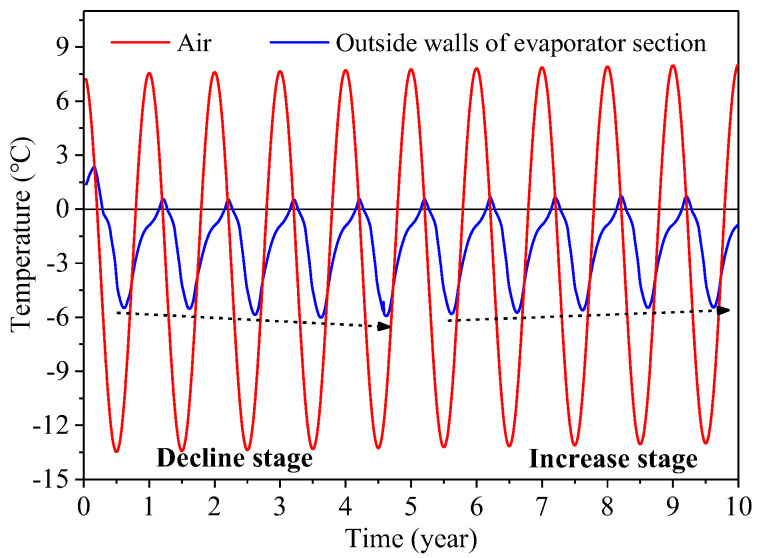
Variations in the average temperatures at the outer walls of the evaporator sections of L-shaped TPCTs with time (α = 50°).

Figure 5 shows the variations of the average temperatures at the outer walls of the evaporator sections of L-shaped TPCTs (*α* = 50°) with time. It can be seen that the temperature is closely related to the air temperature. When the atmospheric temperature is lower than the temperature at the outer walls of the evaporator sections, the L-shaped TPCTs begin to work. With decreases in the air temperature, the cooling performance of the L-shaped TPCTs is improved, and the temperature of the soil around evaporator sections decrease. In the warm season, the L-shaped TPCTs stop working and the temperature of soil around the evaporator sections increase. During the first 5 years, the temperatures at the outer walls of the evaporator sections decrease year by year, reaching a dynamic balance with the ground temperature of the surrounding soil in the fifth year. Then, with increases in the air temperature, it slowly increases.

**Figure 6:**
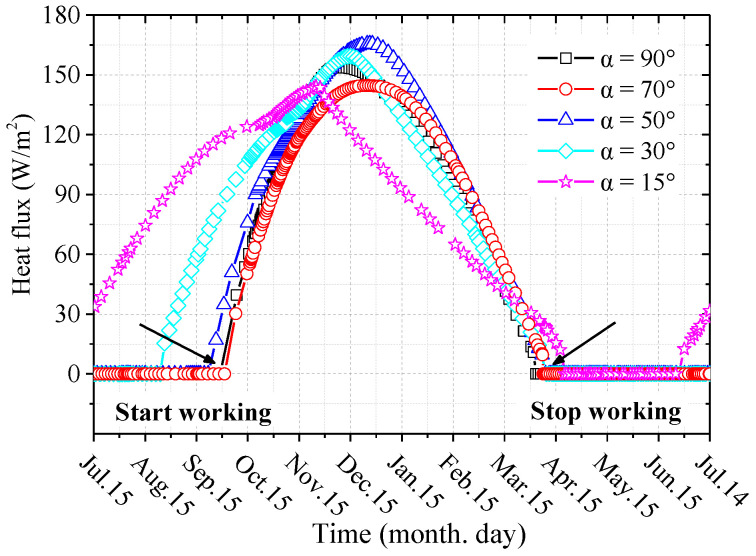
Variations in the heat flux at the outer walls of the evaporator sections of L-shaped TPCTs during the fifth year.

Figure 6 shows the variations of the heat flux at the outer walls of the evaporator sections of L-shaped TPCTs during the fifth year. It can be seen from Figure 5 and Figure 6 that the changes in the heat flux are closely related to the changes in the atmospheric temperature and the outer wall temperatures of the L-shaped TPCT evaporator sections. With decreases in the air temperature, the L-shaped TPCT begins to work, and the heat flux gradually increases. With increases in the air temperature, the heat flux decreases gradually until the L-shaped TPCT stops working. The heat flux is the largest when the inclination of the L-shaped TPCT evaporator section is 50°, reaching the maximum value of 165.7 W·m^−2^ in January. It can also be seen from the figure that the working hours of L-shaped TPCT with different inclinations are also different; a smaller inclination leads to longer working hours.

### 4.2. Geotemperature Distribution in the Subgrade

**Figure 7:**
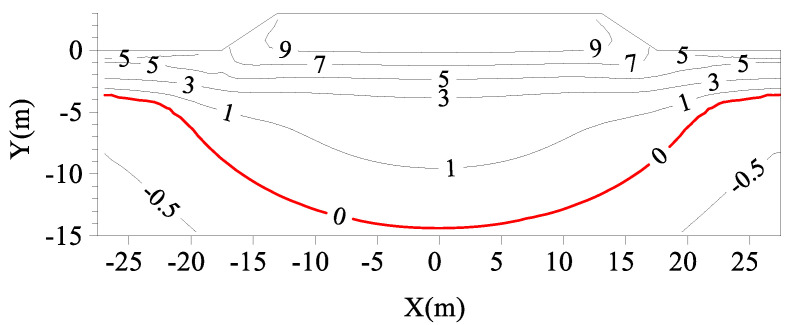
Geotemperature distributions of subgrades without a TPCT on 1 October of the 30th year after construction (Unit: °C).

Figure 7 shows the geotemperature distributions in the subgrade without a TPCT on 1 October of the 30th year after construction. According to the calculation, the permafrost table of natural frozen soil before subgrade construction was 2.86 m. It can be seen from the figure that, within 30 years after the subgrade construction, the permafrost table (0 °C isotherm) at the shoulder decreased from 2.86 m below the ground surface to 11.7 m, and the permafrost table at the center of the subgrade decreased from 2.86 m below the ground surface to 14.37 m, forming a large melting circle for the entire subgrade. Therefore, the wide pavement of the expressway has a strong heat absorption capacity, which leads to the degradation of permafrost, particularly the severe degradation of permafrost at the center of the subgrade.

**Figure 8:**
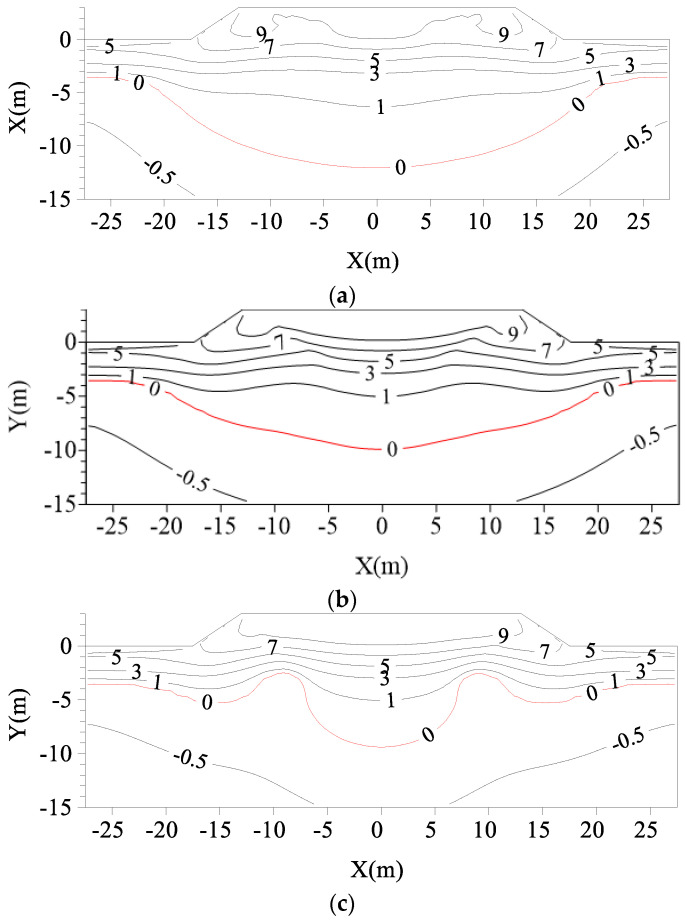

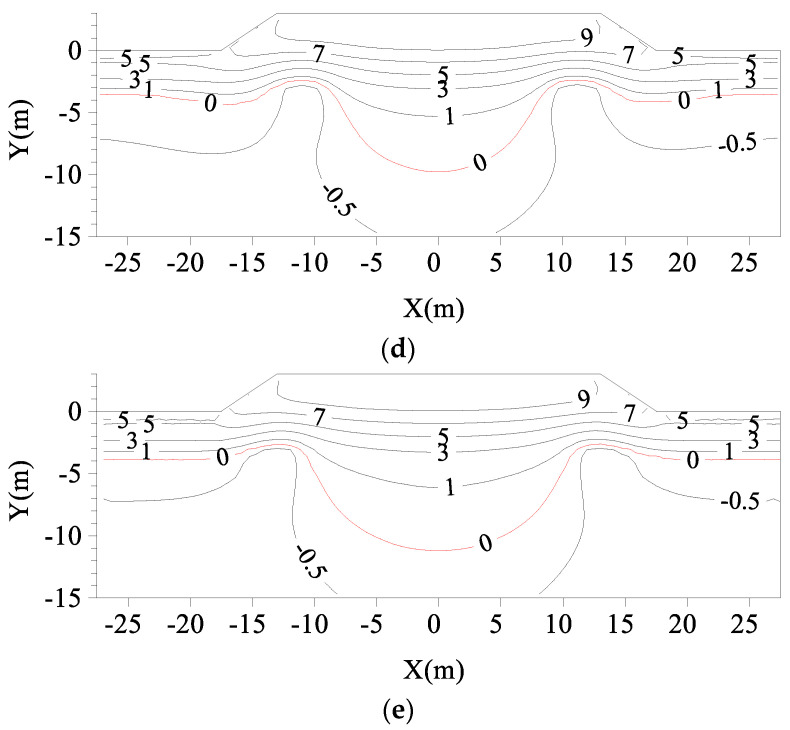
Geotemperature distributions of subgrades with L-shaped TPCTs under different α values on 1 October of the fifth year after their construction (unit: °C): (**a**) α = 15°; (**b**) α = 30°; (**c**) α = 50°; (**d**) α = 70°; (**e**) α = 90°.

Figure 8 shows the geotemperature distributions of subgrades with L-shaped TPCTs under different α values on 1 October of the 30th year after their construction. In comparison to the geotemperature distributions of subgrades without TPCT (Figure 7), the melting circle of the subgrades with L-shaped TPCTs is obviously smaller. It can be seen that the inclination angle of the evaporator section has a great impact on the distribution of the temperature fields of the subgrades. The vertically placed TPCT (Figure 8e) has a good cooling effect on the soil at the shoulder, but has little impact on the temperature at the center of the subgrade.

**Figure 9:**
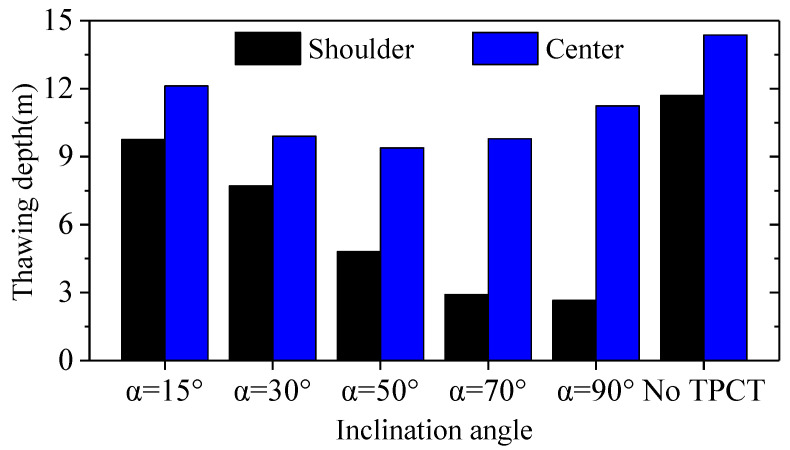
The thawing depth at the subgrade shoulder and center on 1 October after 30 years of construction.

Figure 9 shows the thawing depth at the subgrade shoulder and center 30 years after subgrade construction. It can be seen that, when the inclination angles of the evaporator section are 50° and 70°, the thawing depth at the center of the subgrade is relatively small, and the melting depth is 9.39 m and 9.79 m below the natural ground surface, respectively.

In combination with Figure 6, Figure 8, and Figure 9, considering the heat transfer efficiency of the L-shaped TPCT, the geotemperature distributions of the subgrade, and the thawing depth at the shoulder and center of the subgrade, it can be concluded that the L-shaped TPCTs have relatively good cooling effects on the subgrade as a whole when the inclination angles of the evaporator section are 50° and 70°. However, in the 30th year, the thawing depth at the center of the subgrade with L-shaped TPCTs reaches 9 m below the ground surface; therefore, the L-shaped TPCT cannot be directly applied to the subgrade of a wide expressway alone. It also needs to be used in combination with other thermal insulation measures to ensure the long-term thermal stability of the subgrade.

## 5. Composite Subgrades with an L-Shaped TPCTs/Vertical TPCT/XPS Insulation Board System

**Figure 10:**
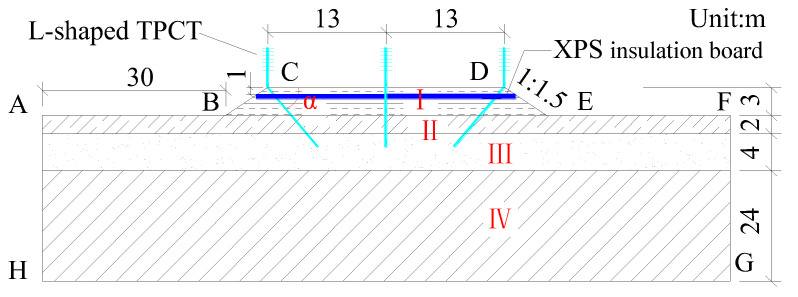
Calculation model of the composite subgrade.

**Figure 11:**
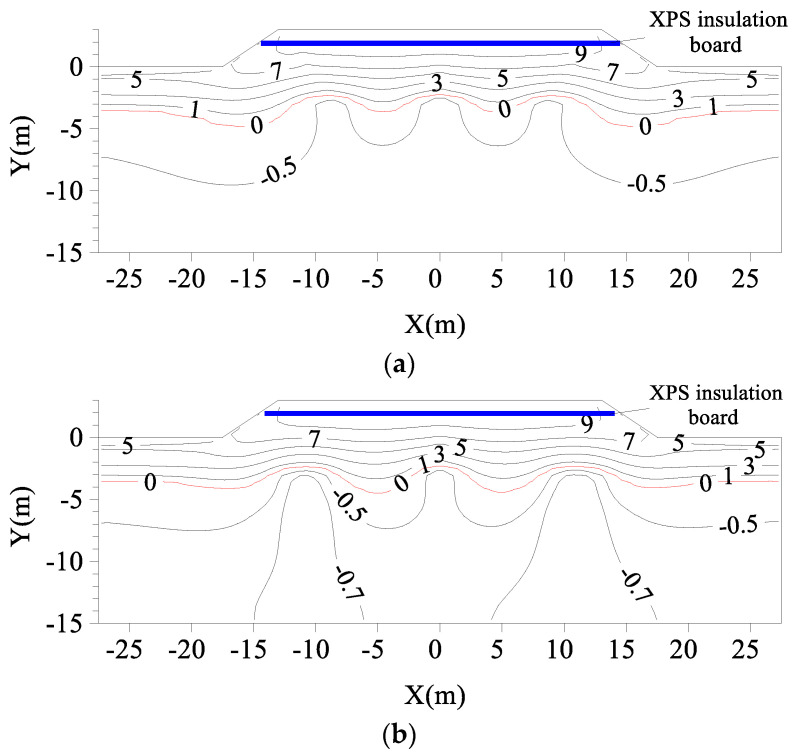
Geotemperature distributions of the composite subgrade on 1 October of the 30th year after their construction (Unit: °C): (**a**) α = 50°; (**b**) α = 70°.

XPS insulation boards are often used as engineering measures to control the ground temperature of frozen foundations, and they can prevent the heat of the asphalt pavement entering the frozen soil foundation during the warm season [34,35,36]. In order to further expand the cooling range and enhance the thermal stability of the wide subgrade, a TPCT is vertically buried at the central reservation of the expressway. The L-shaped TPCTs, vertical TPCT, and XPS insulation board are combined to form a composite subgrade structure. The length of the evaporator section of the vertical TPCT is 6.5 m, and other parameters are the same as the L-shaped TPCTs. XPS insulation boards are set at 1.0 m below the subgrade surface, with a lateral laying width of 29.0 m, as shown in Figure 10. The thickness of the XPS insulation board is 0.10 m, the density is 60 kg·m^−3^, the thermal conductivity is 0.03 W·m^−1^·°C^−1^, the specific heat capacity is 140 J·kg^−1^·°C^−1^, and other calculation parameters and boundary conditions are the same as those in Figure 3. Figure 11 shows the geotemperature distributions of the composite subgrade on 1 October of the 30th year after their construction.

It can be seen from Figure 11 that the composite subgrade increases the permafrost table at the center of the subgrade compared with the geotemperature distributions of the subgrade with L-shaped TPCTs (Figure 8c,d). The permafrost table of natural frozen soil in the 30th year is 3.53 m below the natural ground surface. When α is 50° and 70°, the permafrost table at the center of the composite subgrade is located at 3.64 m and 4.49 m below the natural ground surface, respectively. Therefore, the cooling effect of the composite subgrade is better when α is 50°, and the permafrost table at the center of the composite subgrade is 0.11 m lower than the permafrost table of the natural frozen soil at this time. It can be seen that the composite subgrade with the L-shaped TPCTs/vertical TPCT/XPS insulation board system has a good cooling effect, can resist the warming effect of the climate, and meet the long-term thermal stability requirements of the subgrade in permafrost regions.

**Figure 12:**
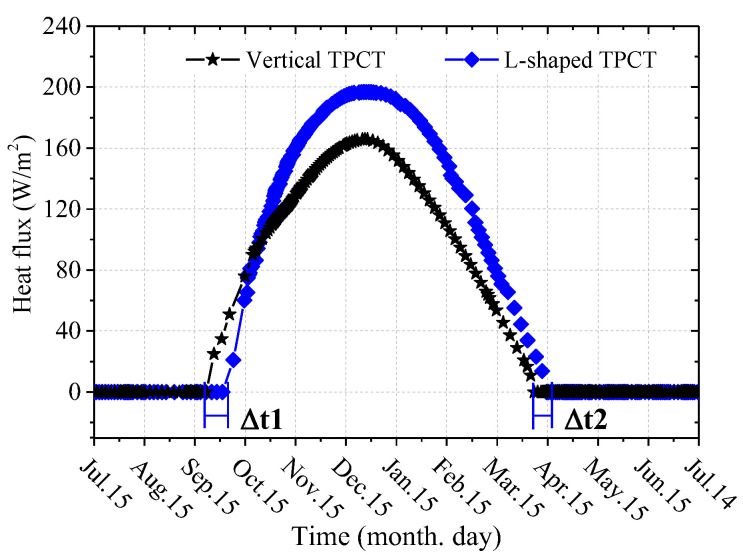
Comparison curves of the heat flux of the vertical TPCT and L-shaped TPCT of the composite subgrade.

Figure 12 shows the comparison curves of the heat flux at the outer wall of the evaporation section of the vertical TPCT and L-shaped TPCT (α is 50°). It can be seen from the figure that the startup time of L-shaped TPCT lags behind, and the corresponding end time also lags behind, where Δt_1_ = 12 days and Δt_2_ = 7 days. During the initial working period of TPCTs, the heat flux of the vertical TPCT of the composite subgrade is slightly higher than that of the L-shaped TPCT. After about 1 month, the heat flux of L-shaped TPCT is greater than that of vertical TPCT. The maximum heat flux of the L-shaped TPCT and the vertical TPCT is 196.8 W·m^2^ and 165.7 W·m^2^, respectively, throughout the working time, and the maximum heat flux of the L-shaped TPCT is 18.8% higher than that of the vertical TPCT. The main reasons for the above phenomena are the different lengths, inclinations, and positions of the evaporation section of TPCTs in the subgrade. In addition, the XPS insulation board exhibits a bidirectional heat resistance effect, which hinders the exchange of heat and cold energy between the atmospheric environment and the subgrade, leading to changes in the temperature difference between the outer wall of the evaporator section of the L-shaped TPCT and the atmospheric temperature, thus affecting the working state of the TPCTs.

## 6. Conclusions

In this paper, a coupled heat transfer model of air temperature–L-shaped TPCT–subgrade was established. The influence of the inclination angles of the evaporator section on the heat-transfer characteristics of the wide subgrade with an L-shaped TPCT in permafrost regions and the thermal stability of the subgrade 30 years later were simulated. The cooling effectiveness of the wide subgrade with L-shaped TPCTs/vertical TPCT/XPS insulation board system was calculated. The main conclusions are as follows:

1. A coupled heat transfer calculation model of air temperature–L-shaped TPCT–subgrade soil, considering the changes in the evaporator section inclination angles of the L-shaped TPCT, was proposed. The comparison between the calculated results and the field-measured data showed that the model could simulate the thermal stability of a wide subgrade with L-shaped TPCTs in permafrost regions.
2. In comparison to a vertical TPCT, the L-shaped TPCT had a relatively good cooling effect on the subgrade, as a whole, when the inclination angles of the evaporator section were 50° and 70°. However, in the 30th year, the thawing depth at the center of the subgrade with L-shaped TPCTs reached 9.0 m below the ground surface. When the evaporator section inclination angle of the TPCT was 50°, the corresponding heat flux was the largest, reaching a maximum value of 165.7 W·m^−2^ in January.
3. The composite subgrade with L-Shaped TPCTs/vertical TPCT/XPS insulation board system is an effective method to protect the permafrost foundation and improve the long-term thermal stability of a wide subgrade. The maximum heat flux of evaporation section of the L-shaped TPCT and vertical TPCT of the composite subgrade was 196.8 W·m^2^ and 165.7 W·m^2^, respectively, throughout the working time, and the maximum heat flux of L-shaped TPCT was increased by 18.8%. However, this paper only studied the influence of L-shaped two-phase closed thermosyphons and XPS insulation boards on the subgrade temperature field, and more studies should be carried out on the deformation of the composite subgrade in the future.

## Figures and Tables

**Table 1:** Physical parameters of air [25].

Parameter	λ_a_ (W·m^−1^·°C^−1^)	*c*_a_ (J·kg^−1^·°C^−1^)	*ρ*_a_ (kg·m^−3^)	*μ* (Pa·s)
Value	0.023	10040	0.641	1.75 × 10^−5^

**Table 2:** Physical parameters of the working medium [29].

Parameter	λ_l_ (W·m^−1^·°C^−1^)	*c_p_*_a_ (J·kg^−1^·°C^−1^)	*ρ*_l_ (kg·m^−3^)	*ρ*_va_ (kg·m^−3^)	*μ* (Pa·s)	*L*_r_ (kJ·kg^−1^)
Value	0.298	2125	638.6	3.48	2.35 × 10^−4^	1263

**Table 3:** Thermophysical parameters of different soils.

Soil Layer	Soil Layer Thickness (m)	Dry Density (kg·m^−3^)	Thermal Conductivity (W·m^−1^·°C^−1^)		Specific Heat Capacity (J·kg^−1^·°C^−1^)		Latent Heat (J·m^−3^)
			Frozen Soils	Unfrozen Soils	Frozen Soils	Unfrozen Soils	
Subgrade fill	3	2060	7128	6908	928	1081	2.04 × 10^7^
Gravelly sand	2	1900	5944	5220	960	1292	2.32 × 10^7^
Clayey loam	4	1600	4864	4050	1174	1473	6.03 × 10^7^
Mudstone	24	1800	6567	5306	1025	1166	3.77 × 10^7^

**Table 4:** Temperature parameters of the upper surface [25].

Variable	*T*_0_ (°C)	*A* (°C)
Air temperature	−3.0	10.5
Asphalt pavement surfaces (CD)	3.5	15
Slope surfaces (BC, DE)	1.7	13
Natural ground surfaces (AB, EF)	−0.5	12

## Data Availability

Not applicable.
